# Oral Infection Caused by *Stenotrophomonas maltophilia*: A Rare Presentation of an Emerging Opportunistic Pathogen

**DOI:** 10.1155/2020/6346120

**Published:** 2020-01-29

**Authors:** Marcelo Prates, Fernando Fernandes, Francisco Proença, Yashad Mussá, Ana Tavares, André Pereira

**Affiliations:** Stomatology Department, Centro Hospitalar Universitário de Lisboa Central ‐ Hospital de São José, Lisbon, Portugal

## Abstract

*Stenotrophomonas maltophilia* is an emerging multidrug-resistant opportunistic pathogen with an increasing incidence of nosocomial and community-acquired infection cases, mainly in immunocompromised individuals. Oral cavity infections are rare. To learn more about this infection, a case of oral cavity infection caused by *S. maltophilia* in an immunosuppressed patient under ventilatory therapy has been presented. The patient presented with multiple nonpainful erosive lesions of the tongue, palate, and oral mucosa. A smear of the oral lesions was performed that revealed the presence of *S. maltophilia* and *Candida albicans*, and the patient was treated with fluconazole and sulfamethoxazole associated with trimethoprim in accordance with the antimicrobial susceptibility testing. After 14 days of antibiotic therapy, there were almost no signs of the previous lesions.

## 1. Introduction

*Stenotrophomonas maltophilia* is an emerging multidrug-resistant opportunistic pathogen [1]. It is a Gram-negative obligate aerobe bacterium found in aqueous habitats. In clinical practice, it is usually found in hospital suction tubing, ventilator inspiratory or expiratory circuits, central venous catheters, dental suction system hoses, hemodialysis water, and dialysate of renal units [1, 2]. Although not highly virulent, the increasing incidence of nosocomial and community-acquired *S. maltophilia* infections is of particular concern for immunocompromised individuals, as this bacterial pathogen is associated with high morbidity and mortality [1–6]. Risk factors for *Stenotrophomonas maltophilia* infection include underlying malignancy [7], the presence of indwelling devices [7], chronic respiratory disease, immunocompromised host [7], prior use of antibiotics [8], and long-term hospitalization or ICU stay [6, 9]. Although infections by *Stenotrophomonas maltophilia* can occur in a lot of different organs and tissues, this agent is commonly found in respiratory tract infections like pneumonia or acute exacerbations of chronic obstructive pulmonary disease. Less frequent infections can occur in soft tissue and skin, bloodstream, urinary tract, surgical-site related infections, endocarditis, meningitis, intra-abdominal infections, and endophthalmitis [10–25]. Oral cavity infections are not described.

## 2. Case Report

A 78-year-old female was hospitalized at an intermediate care unit due to a cardiorespiratory decompensation. The patient had history of high blood pressure associated with hypertensive cardiopathy and cardiac insufficiency, diabetes mellitus type 2, chronic kidney disease under dialysis, and chronic obstructive pulmonary disease with respiratory insufficiency.

After 11 days of noninvasive ventilatory support and due to the sudden onset of oral lesions, she was referred to the Stomatology Department for observation. At this moment, the patient was on day 10 of antibiotic therapy with piperacillin-tazobactam because of a pneumonia caused by *Streptococcus pneumoniae* and under oral fluconazole and topical nystatin since the appearance of the oral lesions, without any improvement.

**Figure 1:**
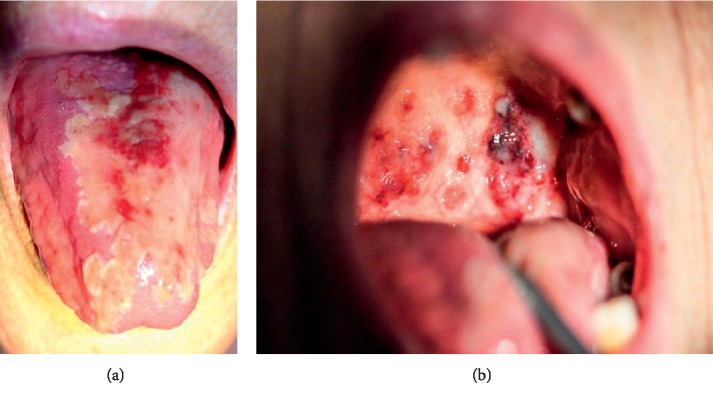
Tongue (a) and palatal (b) lesions when first observed.

Upon clinical examination, there were multiple erosive lesions of the tongue, palate, and oral mucosa (Figure 1). The tongue lesions were covered by a cream color membrane that was detachable. None of the lesions were painful.

We decided to do a biopsy of the ulcers and a smear of the mouth with fungal and bacterial exam and maintain the antibiotic and antifungal therapy.

The histopathological exam was described as an “unspecific ulcer.” The bacterial exam of the smear revealed an infection by a multidrug-resistant *Stenotrophomonas maltophilia* only sensitive to cotrimoxazol (sulfamethoxazole + trimethoprim), and the fungal exam revealed the presence of *Candida albicans*.

Following the antimicrobial susceptibility testing and after discussing with the Intermediate Care Unit team, the antibiotic was changed while maintaining the antifungal therapy.

**Figure 2:**
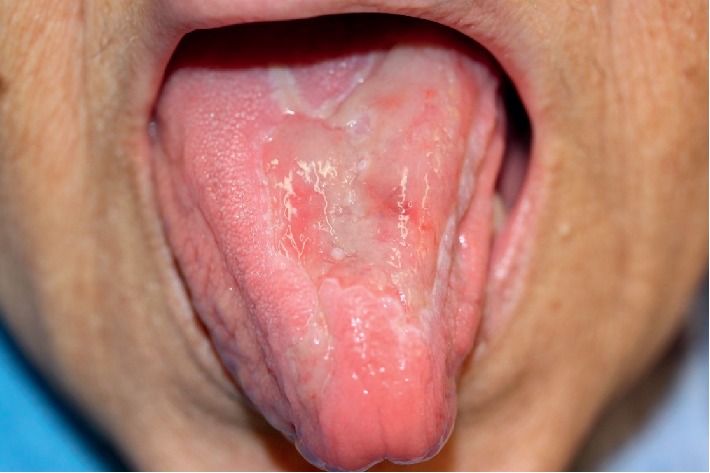
Tongue lesions 6 days after beginning directed antibiotic therapy.

**Figure 3:**
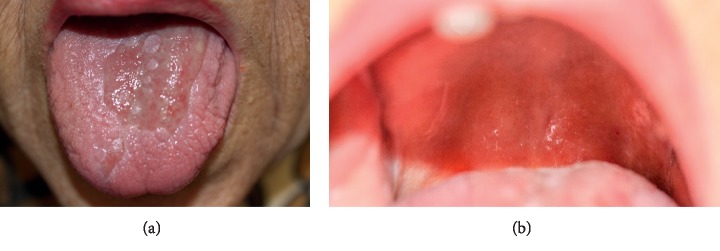
Tongue (a) and palate (b) at the discharge day after 14 days of directed antibiotic therapy.

After 6 days, there were clear signs of improvement (Figure 2). At the day of the discharge, after 14 days of directed antibiotic therapy, there were almost no signs of the previous lesions (Figure 3).

## 3. Discussion and Conclusion

This case report involves a 78-year-old female with an oral infection caused by *Stenotrophomonas maltophilia* and *Candida albicans*. Diagnosis was based on the oral smear with bacterial and fungal exam. Biopsy and histopathological exam were not useful in this case. Confirmation of the infection and the antimicrobial susceptibility test allowed us to change the antibiotic accordingly. The patient had a good oral evolution after beginning directed antibiotic therapy with no prejudice to the improvement of her systemic condition. Due to the fact that *Stenotrophomonas maltophilia* is a multidrug-resistant opportunistic pathogen with an increasing incidence in immunocompromised individuals and since the patient was already under antibiotic therapy, the identification of the agent and its antibiotic susceptibility was a key factor to the positive outcome. This case goes accordingly to literature where it is stated that cotrimoxazol is the most effective antibiotic against *Stenotrophomonas maltophilia* [4, 5, 26–29]. However, there has been an increase in antibiotic resistance observed in *S. maltophilia* isolates worldwide, and the lag in development of new antimicrobials emphasizes the need to develop novel therapeutics [27–31].

In conclusion, the incidence of *Stenotrophomonas maltophilia*-related infections is increasing and we have to be aware of the possibility of oral infections caused by this agent mainly in immunocompromised patients under ventilatory support. This case also shows that the oral smear, which is not usually used in oral cavity due to its rich normal flora, can be an important tool on the diagnosis and treatment of these conditions.
